# AG488 as a therapy against gliomas

**DOI:** 10.18632/oncotarget.18284

**Published:** 2017-05-30

**Authors:** Jadith Ziegler, Anja Bastian, Megan Lerner, Lora Bailey-Downs, Debra Saunders, Nataliya Smith, Jake Sutton, James D. Battiste, Michael A. Ihnat, Aleem Gangjee, Rheal A. Towner

**Affiliations:** ^1^ Advanced Magnetic Resonance Center, Oklahoma Medical Research Foundation, Oklahoma City, OK, USA; ^2^ Department of Pathology, University of Oklahoma Health Sciences Center, Oklahoma City, OK, USA; ^3^ Department of Pharmaceutical Sciences, College of Pharmacy, University of Oklahoma Health Sciences Center, Oklahoma City, OK, USA; ^4^ Department of Surgery Research Laboratory, University of Oklahoma Health Sciences Center, Oklahoma City, OK, USA; ^5^ Stephenson Cancer Center, University of Oklahoma Health Sciences Center, Oklahoma City, OK, USA; ^6^ Graduate School of Pharmaceutical Sciences, Duquesne University, Pittsburgh, PA, USA

**Keywords:** gliomas, magnetic resonance imaging (MRI), *in vivo*, anti-cancer therapy, angiogenesis

## Abstract

High-grade gliomas such as glioblastomas (GBM) present a deadly prognosis following diagnosis and very few effective treatment options. Here, we investigate if the small molecule AG488 can be an effective therapy against GBM with both anti-angiogenic as well as an anti-microtubule inhibiting modalities, using a human G55 glioma xenograft model in nude mice. From *in vitro* studies, we report that AG488 incubation reduced cell viability in G55 and HMEC-1 cells more so than TMZ treatment, and AG488 treatment also decreased cell viability in normal astrocytes, but not as much as for G55 cells (p<0.0001). *In vivo* investigations indicated that AG488 therapy helped reduce tumor volumes (p<0.0001), prolong survival (p<0.01), increase tumor perfusion (p<0.01), and decrease microvessel density (MVD) (p<0.05), compared to untreated mice or mice treated with non-specific IgG, in the G55 xenograft model. Additionally, AG488 did not induce apoptosis in normal mouse brain tissue. Animal survival and tumor volume changes for AG488 were comparable to TMZ or anti-VEGF therapies, however AG488 was found to be more effective in decreasing tumor-related vascularity (perfusion and MVD). AG488 is a potential novel therapy against high-grade gliomas.

## INTRODUCTION

Malignant gliomas are the most common type of brain tumors, and derive from neoplastic glial or neuroglia. Gliomas represent approximately 70% of the 22,500 new cases of malignant primary brain tumors that are diagnosed in the U.S.A. every year [1, 2]. They are classified as either low-grade or high-grade [3]. Patients diagnosed with a high-grade glioma such as glioblastomas (GBMs) are expected to live less than two years after diagnosis [1, 2, 4]. High-grade gliomas are highly vascular, resistant to apoptosis and are very invasive [5]. Standard of care treatments include tumor resection, radiotherapy, chemotherapy, and bevacizumab, an anti-angiogenic agent [6]. Although radiotherapy following tumor resection has been the most effective therapy in the past [7], radiotherapy in conjunction with temozolomide (TMZ), a chemotherapeutic agent, has shown to increase patient survival. Additionally, bevacizumab in conjunction with chemotherapy has been found to be more effective than chemotherapy alone [8–11]. Although these treatments can be effective for some patients, they are toxic and come with short-term or long-term side-effects such as infections, vomiting, fatigue, infertility, and have been associated with secondary forms of cancer [8, 12]. Additionally, radiotherapy and chemotherapy offer no long-term survival and recurrence is almost always the case; therefore, there is a great need for new effective therapies [4, 6, 7, 9].

As high-grade gliomas grow, angiogenesis - the formation of new blood vessels - is a process necessary for tumor growth and invasion [13, 14]. The main pro-angiogenic factors, including vascular endothelial growth factor (VEGF), signal through receptor tyrosine kinases (RTKs) to activate angiogenesis [13, 15]. Although drugs such as the anti-VEGF agent bevacizumab have been effective in inhibiting angiogenesis for gliomas [15, 16], they are not cytotoxic to the cancer cells and have to be combined with cytotoxic chemotherapy such as TMZ. [10, 11]. Additionally, microtubule-targeting agents (MTAs), and microtubule-stabilizing agents (MSAs) are also utilized as chemotherapeutic agents. These molecules are cytotoxic as they disrupt the mitotic spindle that lead to apoptosis [17, 18]. Although effective, these agents can have considerable drawbacks. These include drug resistance, poor CNS penetration, neurotoxicity, negative drug-to-drug interactions, as well as severe cardiac effects; therefore, there is a need for a drug that possesses both anti-angiogenic and cytotoxic properties, and has minimal side-effects [17, 18].

The small molecule AG488 (compound 21 in reference [19]), with the chemical structure [(*N*-(4-Methoxyphenyl)-2,6-dimethyl-*N*-(propan-2-yl)furo [2,3-*d*]pyrumidin-4-amine HCl)], is innovative due to its dual anti-RTK and anti-tubulin mode of action leading to anti-angiogenic and anti-microtubule activities in tumor cells *in vitro* in two murine models of breast cancer [19]. AG488 also resulted in reduced tumor volumes and had lower overall systemic toxicity as compared to the anti-microtubule agent docetaxel and the RTK inhibitor and anti-angiogenic drug sunitinib [19]. This data led us to test whether AG488 could be an effective glioma therapy in an orthotopic human xenograft model, as gliomas are highly vascular and resistant to apoptosis [6].

## RESULTS

**Figure 1:**
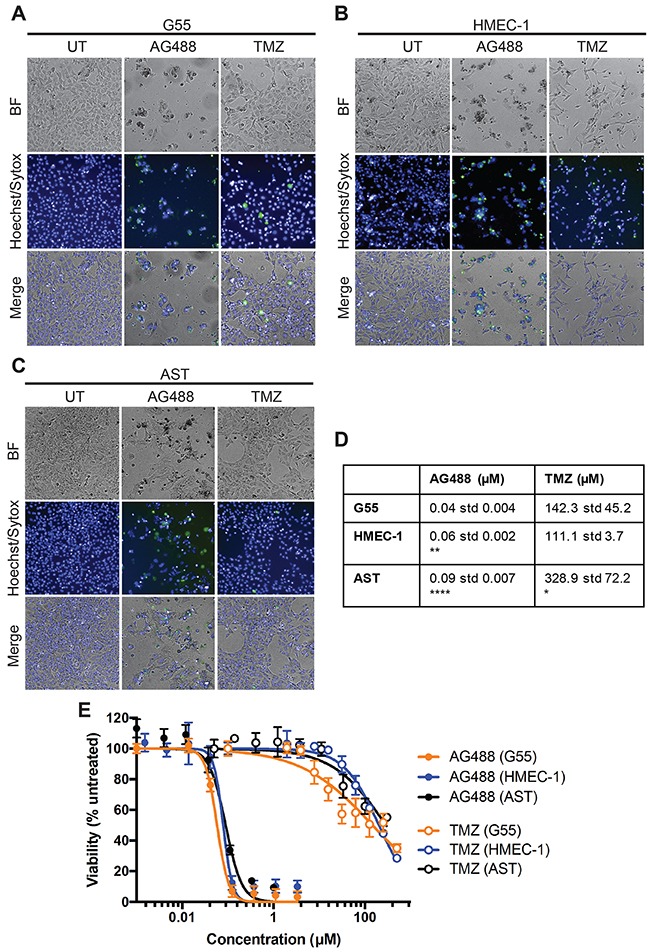
AG488 decreased G55 cell viability G55 or HMEC-1 were seeded in 96-well plates at 3-5 × 10^3^ cells/well. Cells were then incubated with either AG488 (0.12 μM) or TMZ (300 μM). Viable cells were labeled Hoechst (blue) and dead cells were labeled with Sytox (green). Images of G55 cells were obtained **(A)** as well as HMEC-1 **(B)** and AST (primary astrocytes) **(C)**. **(D)** Average EC_50_ values were calculated for each cell line and treatment. Data are listed as means ±S.D. There was a significant difference (*p<0.05, **p<0.01, or ****p<0.0001) when compared to G55 cells. **(E)** Representative dose response curves of G55, HMEC-1 or AST cells treated with either AG488 or TMZ were generated. Data are listed as means ±S.D.

In *in vitro* cell studies, AG488 decreased cell viability of G55 GBM cells, HMEC-1 endothelial cells, and primary astrocytes. Incubation with TMZ also decreased cell viability of all three cell lines. Figure 1A, 1B. EC_50_ (half maximal effective concentration) values were calculated from dose response curves. Figure 1E. Based on the EC_50_ values, astrocytes were significantly less sensitive to AG488 compared to G55 cells, as indicated by the higher EC_50_ values. Similarly, astrocytes and HMEC-1 cells were significantly less sensitive to AG488 compared to G55 cells. Figure 1D. The average EC50 values for AG488 incubated cells were lower than those incubated with TMZ, as shown in the representative dose response curve. Figure 1D, 1E. Data are means ± std. dev.

**Figure 2:**
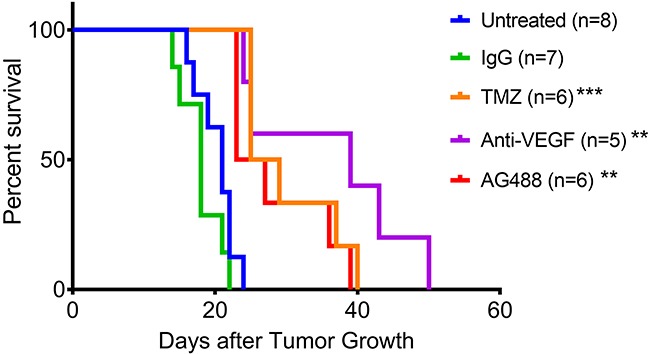
AG488 therapy increases percent survival in G55 gliomas Animal survival curve for G55 glioma-bearing mice either untreated (n=8), IgG treated (n=7), TMZ treated (n=6), anti-VEGF treated (n=5) or AG488 treated (n=6). There was a significant increase in percent survival for all treatment groups (TMZ: p<0.001, anti-VEGF: p<0.01, and AG488: p<0.01) compared to untreated and IgG treated mice (non-specific antibody negative control).

**Figure 3:**
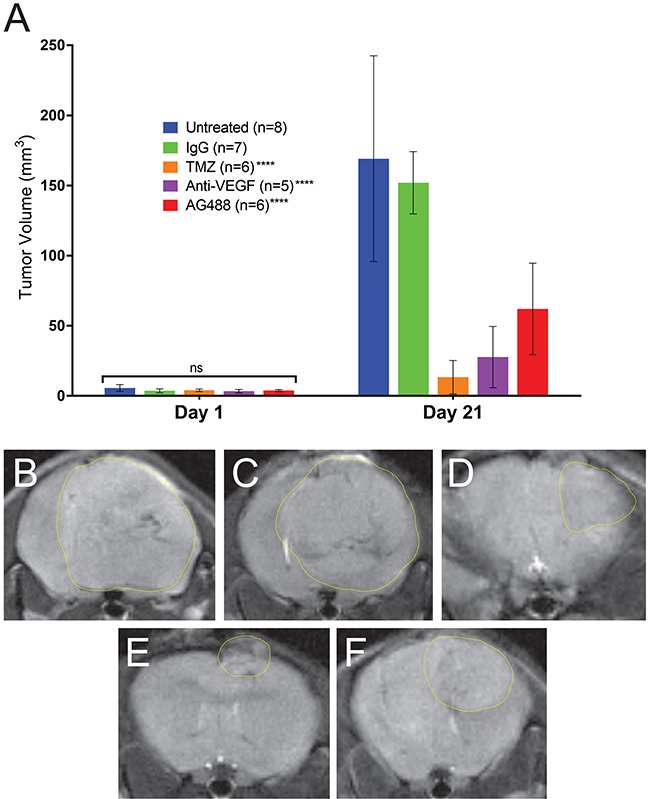
AG488 therapy decreases tumor volumes in G55 gliomas Tumor volumes (mm^3^) were calculated from MR images. **(A)** All treatments had significantly reduced tumor volumes (p<0.0001 for TMZ, anti-VEGF, and AG488) compared to untreated and IgG treated mice. MRI morphological representations (with measured tumor volumes) for each treatment group are depicted in panels for **(B)** untreated (197.94 mm^3^), **(C)** IgG (170.25 mm^3^), **(D)** TMZ (17.31 mm^3^), **(E)** anti-VEGF (20.74 mm^3^), and **(F)** AG488 (31.92 mm^3^) G55 tumor-bearing mice.

**Figure 4:**
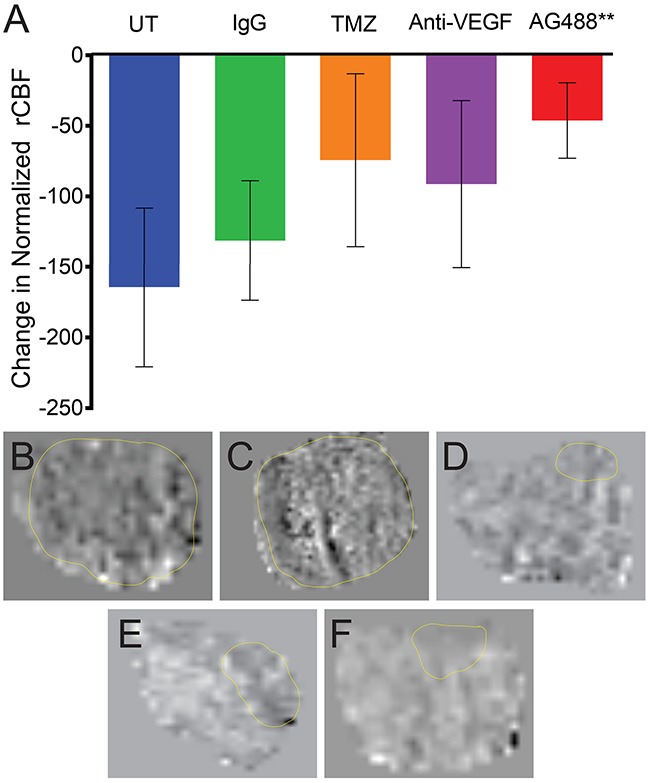
AG488 therapy increases tumor perfusion in G55 gliomas Tumor perfusion was obtained via the MRI technique, arterial spin labeling (ASL), and calculated normalized relative cerebral blood flow (rCBF) values are depicted in a bar graph **(A)**. Treatment with AG488 significantly increased rCBF compared to the untreated and IgG treatment groups (p<0.01). There was no significant change in the TMZ and anti-VEGF treatment groups, compared to the controls. Perfusion maps for each treatment group are depicted for **(B)** untreated, **(C)** IgG, **(D)** TMZ, **(E)** anti-VEGF, and **(F)** AG488 G55 tumor-bearing mice.

**Figure 5:**
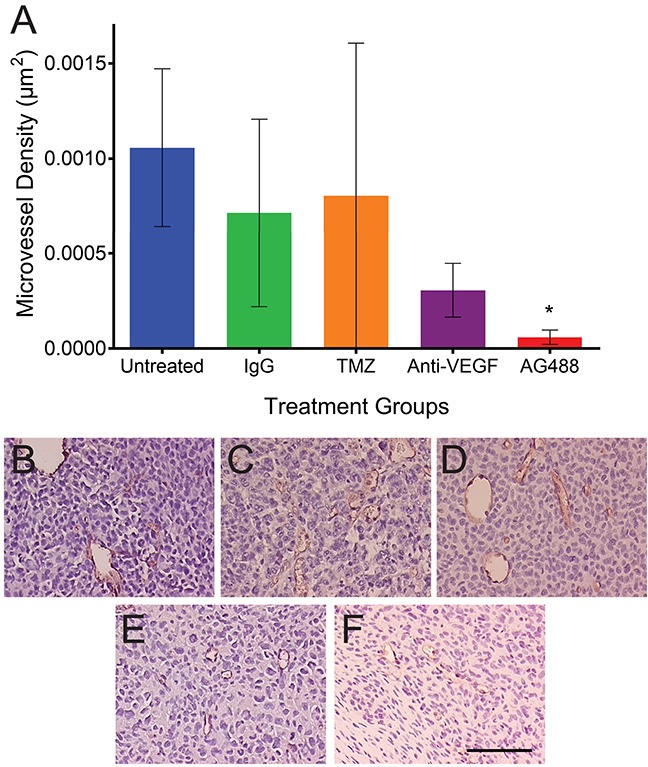
AG488 therapy decreases microvessel density in G55 gliomas Immunohistochemistry was utilized, and tissues were stained for CD34. **(A)** A bar graph depicts AG488 treatment significantly decreased microvessel density in tumor tissue samples, compared to the untreated group (p<0.05). There was no change in the microvessel density for the TMZ and anti-VEGF treatment groups, compared to controls. Histological representative images for each treatment group are depicted for **(B)** untreated, **(C)** IgG, **(D)** TMZ, **(E)** anti-VEGF, and **(F)** AG488 G55 tumor-bearing mice. Scale bar=100 μm. Magnifications are all 20x.

**Figure 6:**
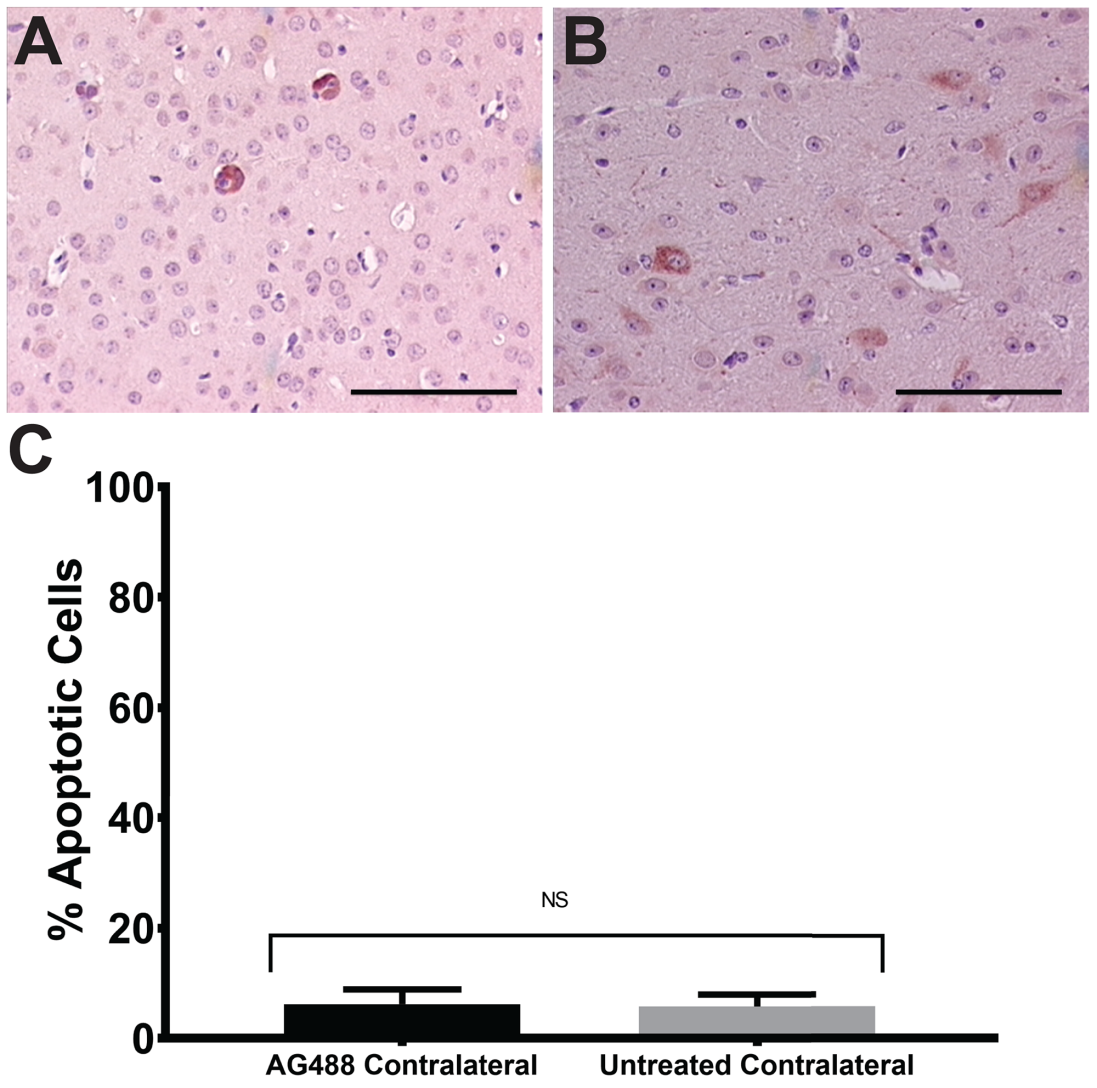
AG488 therapy does not affect normal brain cells Untreated and AG488 treated contralateral tissue was stained with cleaved caspase-3 antibody and apoptosis was assessed. Histological representative images depicted for AG488 contralateral **(A)** and untreated **(B)** tissue. Bar graph in **(C)** depicts no significant difference in % apoptosis between treatment groups. Scale bar=100 μm. Magnifications are all 20x.

From our *in vivo* investigations, it was found that treatment with AG488 significantly increased percent survival for mice compared to the untreated (p<0.01) or IgG-treated (p<0.001) groups. Mice treated with TMZ (p<0.001) and anti-VEGF (p<0.01) also survived longer than the untreated and IgG groups. Figure 2. Twenty-one days after tumor detection, we found that tumor volumes (mean ± S.D.) (32.92±41.19 mm^3^; n=6) for the AG488 group were significantly lower, as well as those treated with the anti-VEGF antibody (15.56±17.17 mm^3^; n=5) and with TMZ (8.72±6.66 mm^3^; n=6) (p<0.0001 for all three agents), compared to untreated (87.42±115.60 mm^3^; n=8) and IgG (77.86±104.90 mm^3^; n=7) treatment groups. Figure 3. We calculated the change in normalized relative cerebral blood flow (rCBF, which measures tumor vascular perfusion) and found that treatment with AG488 significantly increased rCBF compared to the untreated and IgG treatment groups (p<0.01). There was no significant change in normalized rCBF in the TMZ and anti-VEGF treatment groups. Figure 4. CD34 staining revealed that AG488 treatment significantly decreased microvessel density in tumor tissue samples from the untreated group (p<0.05). There was no significant change in the microvessel densities for the TMZ and anti-VEGF treatment groups compared to untreated or IgG-treated groups. Figure 5. Finally, staining for cleaved caspase-3 revealed that there was no significant difference in the percent of normal brain cells undergoing apoptosis between the untreated contralateral and AG488 treated contralateral tissue Figure 6.

## DISCUSSION

Our goal is to find an effective treatment against high-grade gliomas. Our lab previously explored another small molecule, AG119, that contained both DNA synthesis and angiogenesis inhibitory properties. This small molecule was shown to have a therapeutic effect in gliomas in a mouse glioma model [20]. In the current study, we explored the therapeutic effect of another molecule AG488, that was previously synthesized [19]. Using the G55 GBM, HMEC-1 endothelial cells, and primary astrocytes, we tested whether treatment with AG488 would have an effect on cell viabilities. Previous work suggested that AG488 is able to reduce tumor volumes and demonstrate anti-proliferative activity in two murine cancer models [19]. Here, we found that AG488 reduced cell viability for G55 GBM cells more effectively than TMZ. This might be due to the molecule's ability to completely inhibit the polymerization of tubulin, thus disabling the cancer cells’ ability to proliferate [19]. We also found that AG488 did reduce primary astrocyte cell viability, but it was found to be significantly less than with G55 cells. Potentially more relevant to the clinical situation, we also demonstrated that AG488 treatment resulted in no apoptotic cell death in normal cells in mouse brains *in vivo*. The effect of AG488 on the primary astrocytes was possibly due to their high proliferative activity in the cell culture system combined with the anti-proliferative effects of AG488.

Next, we moved to an animal study in an orthotopic G55 xenograft glioma model to confirm the therapeutic effect of AG488. Human G55 was established from a patient GBM, passaged in nude mice, and isolated as a cell line, and subsequently has been used in numerous glioma studies. [21–26]. These cells express EMR2 (epidermal growth factor module-containing mucin-like receptor 2) and retinoblastoma proteins (pRB) [26, 27]. G55 cells have also been found to be more invasive than other glioma cell lines, namely U-87 [28]. In our group, we found that G55 cells migrate at a rate of 420 μm/hour, compared to U-251 cells which migrated at a rate of 8 μm/hour (unpublished data). G55 cells are also TMZ resistant [24]. As a result of the similar patterns in the G55 model in terms of invasiveness/migration, G55 is found to be one of the most physiologically relevant models to date [22, 28]. Additionally, the G55 GBM model is characterized by an increased vascularity and propensity for necrosis, much like tumors found in patients [29].

High-grade gliomas in mice, as well as in patients, have a leaky blood-brain barrier (BBB), which allows small molecules (such as AG488) and other anti-angiogenic therapies to be delivered to the tumor [29–32]. Although this leaky BBB is present in our model, this might not be the case for diffuse GBM, which is very heterogeneous and may have areas of impermeable BBB [5, 33]. Another challenge that we face is that although our xenograft model is an established and relevant model for gliomas, using this mouse model does not replace human samples and does not offer the intratumoral and interpatient heterogeneity that human gliomas possess [34, 35].

Our results indicated that the drug AG488 not only significantly reduced tumor volumes and increased percent animal survival in the human G55 xenograft model, but that AG488 increased tumor vascular perfusion and also decreased microvessel density (MVD) compared to untreated mice. MVD has been used routinely to assess outcomes for treatments, particularly agents affecting angiogenesis; where a higher MVD is correlated with a shorter survival [36–38]. Furthermore, MRI perfusion imaging is a valuable and noninvasive tool, and can also assess anti-angiogenic responses from therapies used for gliomas [36, 39]. AG488 was more effective as an anti-angiogenic agent compared to bevacizumab, as illustrated by the MRI perfusion, as well as MVD data. AG488 has a vascular normalization effect on the tumor tissue, which would be important regarding restoring blood vessel architecture to normal, as well as allow better drug distribution to the tissue. The perfusion data suggests that this method might be a better tool to assess vascular changes after drug treatments. Although TMZ and anti-VEGF results appeared to show a higher percent survival compared to AG488, there was no statistical difference between these individual groups in terms of survival and tumor volumes. Furthermore, antibody treatment against VEGFR2/VEGF although leads to a decrease in tumor volumes, can also cause an increase in tumor invasiveness along the host microvasculature [22, 28, 29]. This suggests that anti-angiogenesis drugs should also be best combined with an anti-tumor drug [22].

This data confirms previous analysis that describes that this small molecule as not only having antitumor properties, but also affecting angiogenesis through the inhibition of RTK's in glioma models, although the precise mechanism by which it is disrupted is unknown [19]. Previous studies have found that RTK inhibitors work best in conjunction with other RTK inhibitors such as sunitinib and gefitinib [targeting Epidermal Growth Factor Receptor (EGFR)] as more pathways are targeted for inhibition [40]. Thus, AG488 should be considered as an effective potential therapeutic agent against high-grade gliomas in combination with an antitumor agent such as TMZ, an anti-angiogenic drug such as Bevacizumab, or an RTK inhibitor.

## MATERIALS AND METHODS

### Cell culture

G55 cells (provided by Michael E. Sughrue, M.D. from the University of Oklahoma, Department of Neurosurgery) were cultured in DMEM (LifeTechnologies, Waltham, MA) supplemented with 10% cosmic calf serum (CCS; HyClone, Logan, UT) and 1 % penicillin/streptomycin. Noncancerous human endothelial microvascular cells (HMEC-1) were obtained from ATCC and cultured in Media-199 (SigmaAldrich) supplemented with 15% fetal bovine serum (VWR, Radnor, PA) and penicillin/streptomycin. Mouse Astrocytes, obtained from ATCC were cultured in DMEM (LifeTechnologies, Waltham, MA), and supplemented with 10% cosmic calf serum (CCS; HyClone, Logan, UT).

### Cell viability assay

Cells were seeded in 96-well plates at 3-5 × 10^3^ cells/well and allowed to attach overnight. AG488 stock was prepared in DMSO and TMZ stock was prepared in OptiMEM and diluted in reduced-serum OptiMEM (LifeTechnologies) before adding to the cells. After four hours of treatment, 10 μL CCS or 15 μL FBS were added to mouse astrocytes, G55 cells or HMEC-1 cells, respectively. Cells were incubated for an additional 44 hours. Cell viability was assessed by labeling all nuclei with Hoechst 33342 (2 μg/ml; Life Technologies) and dead cells with SytoxGreen (0.5 μM; LifeTechnologies). Three images per well were obtained using Operetta High-Content Imaging System (PerkinElmer, Waltham, MA) and used to determine the number of live cells per field. Nonlinear regression variable slope dose response analysis using Prism 6.0 software (GraphPad, San Diego, CA) was performed to obtain EC_50_ and Hillslope values.

### Mice and treatments

Animal studies were conducted in accordance to the OMRF and OUHSC IACUC policies, which follow NIH guidelines. Two-month-old male nude mice (Hsd:Athymic Nude-Foxn1nu mice; Harlan Inc., Indianapolis, IN) were implanted intracerebrally with human G55 xenograft cells (1 × 10^6^) per mL suspended in 4 μL in cell culture media of 1% agarose solution. Once tumors reached 10-15 mm^3^ (determined via MRI), mice were either treated every 3 days with AG488 or TMZ, both at 30 mg/kg. AG488 was dissolved in 5% N-methylpyrrolidine (Pharmasolve; Sigma-Aldrich), 5 % solutol-15 (BASF, Bern, Switzerland) in sterile normal saline and administered via intraperitoneal (IP) injections. TMZ was dissolved in 5% DMSO and 5% solutol-15 in sterile saline and administered via gavage. Mice were treated until the tumor reached 100-150 mm^3^ or for a total of 4 weeks. Human Anti-VEGF (bevacizumab; Avastin, Genentech), or nonspecific mouse immunoglobulin (Ig)G (Alpha Diagnostics) was also administered at 2 mg/kg in sterile saline via tail vein injections every 2-3 days.

### MRI

MRI experiments were performed on a Bruker Bio-spec 7.0 Tesla/ 30-cm horizontal-bore magnet imaging system. Animals were immobilized by using 1.5–2.5% isoflurane and 0.8 L/min O_2_ and placed in a 72-mm quadrature volume coil for signal transmission, and a surface mouse-head coil was used for signal reception. T2-weighted morphological imaging was obtained with a slice thickness of 0.5 mm, a FOV of 2 × 2 cm^2^ for an approximate in-plane resolution of 80 μm and with a repetition time (TR) of 3000 ms and an echo time (TE) of 63 ms, for a total acquisition time of 13 min. Tumor volumes were calculated from 3D MRI slices rendered MRI datasets, using Amira v5.6.0 (FEI). [41, 42]

### Perfusion imaging

In order to assess microvascular alterations associated with tumor capillaries, the perfusion imaging method, arterial spin labeling (ASL), was used as previously described [43, 44]. Perfusion maps were obtained on a single axial slice of the brain located on the point of the rostro-caudal axis where the tumor had the largest cross-section. Five regions of interest (ROIs) were manually outlined around the tumor, and appropriate ROIs were also taken from the contralateral side of the brain for comparison purposes. To calculate the differences in rCBF values, tumor rCBF values were obtained at late (days 18-26 following intracerebral implantation of cells for untreated mice) and early (days 10-13 following cell implantation) tumor stages, and normalized to rCBF values in the contralateral brain region of corresponding animals. Tumor volumes were transposed from morphological image data sets.

### Histology and immunohistochemistry (IHC)

All mice were euthanized after the last MRI examination. The brain of each animal was removed, preserved in 10% neutral buffered formalin, and processed routinely. Paraffin-embedded tissues were sectioned in 5 μm sections, mounted on super frost plus glass slides, stained with hematoxylin and eosin (H&E), and examined by light microscopy. To characterize microvessel density (MVD) in both untreated and treated groups, immunohistochemistry for anti-CD34 antibody (rabbit anti-CD34, ab 81289, 10 μg/mL, Abcam, Cambridge, MA) was performed. To calculate cells undergoing apoptosis, contralateral AG488 and untreated slides were stained with a cleaved caspase-3 antibody (anti-procaspase 3, sc-7148, 5 μg/mL, Santa Cruz Biotechnology Inc., CA, USA,). Three regions of interest (ROI) in each group were identified, and apoptosis measurements were captured digitally for each selected ROI. They were then analyzed as well as imaged using Aperio ImageScope (Leica Biosystems, Buffalo Grove, IL).

### Statistical analysis

Survival curves were analyzed using Kaplan-Meier curves. Tumor volumes, changes in normalized rCBF, microvessel densities, tumor blood volumes, and cleaved caspase-3 were analyzed and compared by two-way ANOVA with multiple comparisons. Data were represented as mean ± SD, and P-values of either *0.05, **0.01, ***0.001, and ****0.0001 were considered statistically significant.

## Abbreviations

ASL: Arterial Spin Labeling
BBB: blood-brain barrier
CNS: central nervous system
DMEM: Dulbecco's Modified Eagle's Medium
DMSO: Dimethyl Sulfoxide
EC_50_: Half Maximal Effective concentration
EGFR: Epidermal growth factor receptor
EMR2: epidermal growth factor module-containing mucin-like receptor 2
GBMs: Glioblastomas
HMEC-1: Human Endothelial Microvascular Cells
IHC: Immunohistochemistry
MRI: Magnetic Resonance Imaging
MSAs: microtubule-stabilizing agents
MTAs: Microtubule-Targeting Agents
MVD: Microvessel Density
pRB: retinoblastoma protein
rCBF: Regional Cerebral Blood Flow
ROIs: Regions of Interest
RTKs: Receptor Tyrosine Kinases
TMZ: Temozolomide
U.S.A.: United States of America
VEGF: Vascular Endothelial Growth Factor
VEGFR2: Vascular Endothelial Growth Factor Receptor 2
